# Ultra-Hypofractionated Whole-Breast Irradiation With or Without Simultaneous Integrated Boost Using Helical Tomotherapy for Early-Stage Breast Cancer: A Real-World Dosimetric and Clinical Outcome Study

**DOI:** 10.3390/cancers18061015

**Published:** 2026-03-20

**Authors:** Pei-Yu Hou, Chen-Hsi Hsieh, Hsin-Pei Yeh, Eva Yu-Hsuan Chuang

**Affiliations:** 1 Division of Radiation Oncology, Department of Radiology, Far Eastern Memorial Hospital, New Taipei City 220216, Taiwan; 2 Department of Computer Science and Engineering, Yuan Ze University, Taoyuan 320315, Taiwan; 3 School of Medicine, College of Medicine, National Yang Ming Chiao Tung University, Taipei 112, Taiwan

**Keywords:** breast cancer, ultra-hypofractionation, whole-breast irradiation, Helical Tomotherapy, simultaneous integrated boost, dosimetry

## Abstract

**Background**: Ultra-hypofractionated whole-breast irradiation (WBI) delivering 26 Gy in five fractions has been established as a standard of care following the FAST-Forward trial. However, real-world data addressing advanced delivery techniques and the feasibility of incorporating a simultaneous integrated boost (SIB) remain limited. **Methods**: We retrospectively analyzed 40 patients with early-stage breast cancer (pT1–2N0M0) treated with breast-conserving surgery, followed by ultra-hypofractionated WBI using helical tomotherapy. Patients received either WBI alone (26 Gy in five fractions) or WBI with an SIB to the tumor bed (29–30 Gy in five fractions). Dosimetric parameters for planning target volumes (PTVs) and organs at risk (OARs) were evaluated. Acute skin toxicity was assessed using CTCAE version 5.0. **Results**: The median patient age was 55.7 years. The mean PTV V95% was 97.8%, with excellent hotspot control (PTV V105% < 5% and V107% < 2%). For left-sided tumors, the mean heart dose was 1.67 Gy, and the ipsilateral lung V8Gy remained below 15% in all patients. Acute radiation dermatitis was limited to Grade 0–1 in all cases. At a median follow-up of 14.8 months, both local control and overall survival were 100%. **Conclusions**: Ultra-hypofractionated WBI delivered using helical tomotherapy, with or without SIB, demonstrates robust dosimetric quality, minimal acute toxicity, and favorable early clinical outcomes in routine clinical practice.

## Simple Summary

Ultra-hypofractionated whole-breast irradiation (WBI) has become a standard of care for selected patients following the FAST-Forward trial. This study evaluated the real-world application of this regimen using helical tomotherapy, specifically focusing on the feasibility of adding a simultaneous integrated boost (SIB) to the tumor bed. Results from 40 patients with early-stage breast cancer showed that this advanced delivery technique provides high-precision dose delivery with excellent protection of the heart and lungs. Clinical outcomes were favorable for local control and skin toxicity. The findings suggest that ultra-hypofractionated WBI via helical tomotherapy, with or without SIB, is a safe and effective treatment option in routine clinical practice.

## 1. Introduction

Adjuvant whole-breast irradiation (WBI) following breast-conserving surgery is a cornerstone of management for early-stage breast cancer. For decades, adjuvant radiotherapy has played a critical role in reducing local recurrence and improving survival after breast-conserving surgery. Historically, conventional fractionation schedules delivering 50–50.4 Gy in 25–28 fractions over five to six weeks were regarded as the standard of care; however, such prolonged treatment courses impose substantial logistical, socioeconomic, and healthcare resource burdens.

Over the past two decades, hypofractionated radiotherapy has fundamentally reshaped breast cancer radiotherapy practice. Large randomized trials from Canada, the United Kingdom START A and START B, and the DBCG HYPO Trial demonstrated that moderate hypofractionation delivered over 15–16 fractions provides equivalent tumor control with comparable or improved toxicity profiles relative to conventional fractionation [1,2,3,4,5,6]. These findings prompted further investigation into whether treatment duration could be shortened even further without compromising oncologic efficacy or normal tissue safety.

The UK FAST trial represented a pivotal radiobiological milestone by evaluating once-weekly five-fraction whole-breast radiotherapy schedules [7]. With long-term follow-up approaching 10 years, the FAST trial demonstrated that 28.5 Gy delivered in five fractions achieved late normal tissue effects and local tumor control comparable to conventional fractionation, whereas higher per-fraction doses (30 Gy in five fractions) were associated with increased toxicity. These results helped define the upper limits of fraction-size sensitivity in breast tissue and provided important α/β estimates supporting accelerated fractionation [8].

Building upon this foundation, the FAST-Forward phase III randomized non-inferiority trial translated these radiobiological insights into a clinically practical one-week treatment paradigm. At five years, 26 Gy delivered in five daily fractions was shown to be non-inferior to 40 Gy in 15 fractions with respect to ipsilateral breast tumor relapse, while maintaining comparable late normal tissue effects [9,10,11]. As a result, ultra-hypofractionated whole-breast irradiation has been increasingly adopted for selected patients, supported by contemporary guideline statements emphasizing improved patient convenience and more efficient use of healthcare resources [12].

Despite these advances, the implementation of ultra-hypofractionated radiotherapy in real-world practice raises several important considerations. First, dose homogeneity and hotspot control are particularly critical in five-fraction regimens, given the steep dose–response relationship of breast tissue. Second, concerns persist regarding organ-at-risk exposure, especially cardiac and pulmonary doses, when modern intensity-modulated and rotational techniques are employed. Third, while tumor-bed boost irradiation has traditionally been delivered sequentially, incorporation of a simultaneous integrated boost (SIB) into an ultra-hypofractionated regimen offers substantial logistical advantages but requires careful validation of dosimetric feasibility and clinical safety [13].

Helical tomotherapy is a rotational, image-guided intensity-modulated radiotherapy platform capable of delivering highly conformal dose distributions across complex breast geometries. Its potential advantages include improved target coverage, enhanced dose homogeneity, and precise daily image guidance. However, clinical outcome and toxicity data specifically addressing ultra-hypofractionated adjuvant breast radiotherapy delivered using helical tomotherapy remain limited.

Therefore, the present study aims to report real-world clinical outcomes, dosimetric quality, and acute toxicity associated with ultra-hypofractionated WBI delivered using helical tomotherapy, with or without SIB, at a single institution. We specifically evaluate the feasibility of incorporating an SIB (29–30 Gy in five fractions) while maintaining stringent organ-at-risk sparing and target coverage.

## 2. Materials and Methods

### 2.1. Patient Population

This retrospective study included 40 consecutive female patients with pathologically confirmed early-stage breast cancer (invasive ductal carcinoma, invasive lobular carcinoma, or ductal carcinoma in situ) who were receiving adjuvant RT at Far Eastern Memorial Hospital (FEMH) in Taiwan between January 2024 and June 2025. Eligibility criteria were as follows: patients who underwent breast-conserving surgery, had pathological stage pTis–3N0M0 disease, and received adjuvant ultra-hypofractionated whole-breast irradiation (WBI) delivered using helical tomotherapy. While the eligibility criteria for the FAST-Forward trial included pT1–3, pN0–1, and M0 status following breast-conserving surgery (BCS) or mastectomy, our study focused on early-stage breast cancer without lymph node involvement. Consequently, we limited our inclusion criteria to patients who underwent BCS and were pN0.

Patients with distant metastases, prior thoracic irradiation, or indications for regional nodal irradiation were excluded. Clinical, pathological, and treatment-related data were retrieved from institutional medical records. This study received approval from the Human Experimentation Committee of FEMH (FEMH-114175E).

### 2.2. RT Treatment Plan

All patients underwent computed tomography (CT) simulation in the supine position with a 2.5 mm slice thickness using a Discovery CT590 RT scanner (GE Healthcare, Chicago, IL, USA). The resulting images were used for RT treatment planning to delineate the target volumes and adjacent OARs. CT simulation was performed under free-breathing conditions. The clinical target volume (CTV) encompassed the entire breast, as defined by institutional contouring guidelines. Patients were selected for SIB based on institutional protocols adapted from European Society for Radiotherapy and Oncology (ESTRO) guidelines for boost [14]. Criteria included: positive/close margins (strongly recommend), Grade 3 (optional), or age ≤ 50 (optional). The tumor bed CTV (CTV_boost) was delineated by the surgical cavity, ideally identified by surgical clips, surrounding soft tissue change, consistent with the seroma seen on imaging. To account for setup uncertainties, the planning target volume (PTV) was generated by applying a 5–6 mm expansion to the CTV. Treatment accuracy was ensured through daily CT-based image guidance, which allowed for precise verification of the internal anatomy and soft tissue localization. The prescribed dose for whole-breast irradiation was 26 Gy delivered in five fractions (5.2 Gy per fraction). Patients with higher-risk features received a simultaneous integrated boost (SIB) to the tumor bed, delivering a total dose of 29–30 Gy (5.8–6.0 Gy per fraction) in five fractions. The SIB regimen was informed by two primary sources. First, the ongoing FAST-Forward Boost trial [13], which investigates one group with WBI dose of 26 Gy in 5 fractions and a 30 Gy SIB to the tumor bed. Second, Dzhugashvili, M., et al. reported comparable acute toxicity using a 29 Gy SIB [15]. To compare our 5-fraction SIB to a standard 40 Gy/15-fraction WBI plus 10 Gy/5-fraction sequential boost, we accounted for a repopulation ‘time penalty’ of 0.6 Gy per day for treatment courses exceeding 21 days. Consequently, a 3.0 Gy penalty was applied to the sequential regimen (26 days total). To mitigate the risk of late-term fibrosis associated with high dose-per-fraction delivery, a SIB dose of 29–30 Gy was selected. This regimen provides a slightly lower biologically effective dose (BED) than a sequential boost of 10 Gy in 5 fractions, based on a presumed α/β ratio of 4.0 Gy for breast tissue.

### 2.3. RT Technique and Dosimetric Evaluation

All treatments were delivered using helical tomotherapy (HT) with daily image-guided radiotherapy (IGRT) to ensure accurate target positioning and reproducibility. Dosimetric evaluation focused on PTV coverage, hotspot control, and organ-at-risk (OAR) sparing. Planning objectives were based on the FAST-Forward protocol and institutional standards. Target volume constraints were defined as follows: PTV V95% ≥ 95% and maximum dose (D_max) < 107%. Cardiac and pulmonary dose constraints included heart V1.5Gy < 30% and heart V7Gy < 5%, as well as ipsilateral lung V8Gy < 15%. The Supplementary Table S1 provides comprehensive details on the optimization constraints utilized in our institutional protocols.

HT plans were optimized to enhance target coverage homogeneity and conformity while limiting the dose to adjacent OARs, particularly the heart and lungs. Dosimetric evaluation was conducted using dose–volume histograms (DVH) to ensure all target and OAR constraints were met. Treatment planning was performed with the TomoTherapy Hi-Art system (v. 5.1.3; Accuray Inc., Madison, WI, USA), and treatments were delivered using either a Hi-Art or HD TomoTherapy platform.

### 2.4. Outcome Assessment and Follow-Up

The primary endpoint of this study was dosimetric quality. Secondary endpoints included local recurrence, overall survival, and acute skin toxicity. Skin care management consisted of the twice-daily application of fragrance-free, aqueous-based moisturizers. The use of prophylactic topical steroids during the treatment was optional. To minimize skin toxicity, no bolus was utilized for the patients in this cohort. Follow-up was calculated from the RT completion date to the date of the last follow-up. Acute radiation dermatitis was assessed using the Common Terminology Criteria for Adverse Events (CTCAE), version 5.0. Patients were routinely followed every week during RT, and 2 weeks and 1 month after completion of RT.

Generative artificial intelligence (GenAI), including Gemini and ChatGPT, were utilized for grammar refinement, data processing, reference searching, and initial drafting of the Discussion framework. The authors have meticulously reviewed, verified, and revised all content to ensure scientific accuracy and integrity.

## 3. Results

### 3.1. Patient Characteristics and Dosimetric Analysis

The median patient age was 55.7 years (with a range of 35–80 years). Most patients presented with T1–T2 tumors and hormone receptor-positive disease (Table 1). Ten patients (25%) received a simultaneous integrated boost. Helical tomotherapy achieved high conformity and homogeneity across a wide range of breast volumes (405–1574 mL). The average beam-on time for our cohort was 19.8 min. Dosimetric parameters for target volumes and organs at risk are summarized in Table 2.

Excellent target coverage was achieved across the cohort. The mean PTV V95% was 97.8%, indicating robust coverage of the prescribed dose. High-dose regions were well controlled, with PTV V105% and V107% remaining below clinically relevant thresholds.

Despite the rotational nature of Tomotherapy, low-dose spillage was effectively controlled, with cardiac and pulmonary doses remaining within predefined constraints. For left-sided breast cancer patients (N = 22), the mean heart dose was strictly controlled (1.67 Gy), minimizing the risk of late cardiac toxicity. The low-dose spillage (V1.5Gy) was maintained below the 30% constraint in all cases. Ipsilateral lung V8Gy was maintained below 15% for all patients.

**Figure 1:**
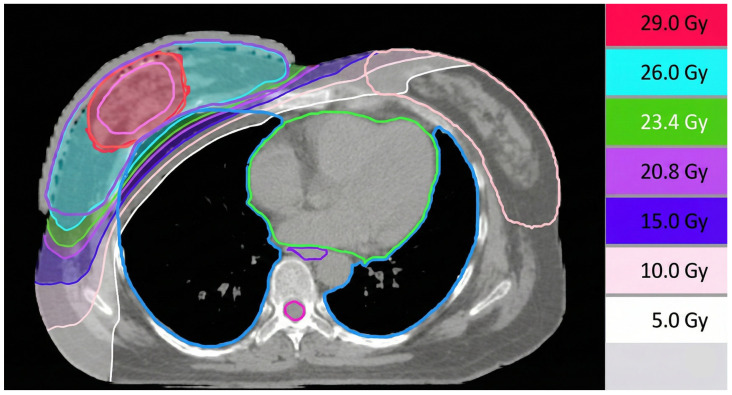
The dose distribution of tomotherapy planning for a case delivering 26 Gy in 5 fractions to the whole breast and simultaneous integrated boost 29 Gy to the tumor bed.

Overall, target coverage remained consistent between the whole-breast-only and SIB cohorts (Table 3). The dose distribution of tomotherapy planning for a case delivering 26 Gy in five fractions to the whole breast and SIB 29 Gy to the tumor bed was shown as Figure 1.

### 3.2. Clinical Outcomes and Acute Toxicity

At a median follow-up of 14.8 months (with a range of 1.1–22.7 months), local control and overall survival were both 100%. Acute skin toxicity was minimal: 50% of patients experienced no skin reaction (Grade 0), and 50% experienced faint erythema (Grade 1). No cases of moist desquamation or Grade ≥ 2 dermatitis were observed (Table 4).

Importantly, patients receiving SIB, including those treated with up to 6.0 Gy per fraction to the tumor bed, did not experience higher grades of acute toxicity compared with patients receiving whole-breast irradiation alone. No cases of symptomatic acute pneumonitis or rib fractures were observed during follow-up.

## 4. Discussion

### 4.1. Comparison with FAST-Forward and Real-World Evidence

This real-world cohort demonstrates that ultra-hypofractionated WBI delivered using helical tomotherapy achieves excellent dosimetric quality with minimal acute toxicity, consistent with randomized evidence supporting five-fraction regimens. Our findings demonstrate that this approach is well tolerated, achieves good target coverage with stringent hotspot control, and is associated with favorable early clinical outcomes. While FAST-Forward predominantly employed three-dimensional conformal techniques, our results extend these landmark findings to an image-guided intensity-modulated delivery platform that helical tomotherapy enabled excellent dose homogeneity and near-complete suppression of high-dose hotspots. This is particularly relevant in five-fraction regimens, which lie on a steep dose–response curve where even modest dose heterogeneity may translate into clinically meaningful toxicity.

Observational studies and institutional cohorts have similarly confirmed the feasibility and safety of ultra-hypofractionated WBI in routine practice, reporting low rates of acute skin toxicity and favorable cosmetic outcomes [16]. Meta-analyses further indicate no significant increase in acute or late toxicity compared with moderate hypofractionation, although continued long-term follow-up remains essential [17].

Recently reported 10-year data from the FAST-Forward trial, summarized in the DEGRO expert statement, provide important reassurance regarding long-term oncologic safety. The 10-year cumulative ipsilateral breast tumor relapse rate for 26 Gy in five fractions was numerically lower than that for 40 Gy in 15 fractions, with comparable breast cancer-free interval and overall survival. Importantly, late normal tissue effects—including breast induration, edema, rib fracture, pulmonary fibrosis, and ischemic heart disease—remained similar between regimens, with no evidence of late divergence [18].

Although follow-up in the present study is shorter, the absence of acute Grade ≥ 2 toxicity and the excellent dosimetric homogeneity achieved with helical tomotherapy are consistent with these long-term safety signals. This supports the DEGRO conclusion that strict hotspot control is critical when implementing ultra-hypofractionated schedules.

The FAST-Forward randomized nodal sub-study further provides critical context for treatment selection. At five years, patient- and clinician-reported arm and shoulder morbidity following axillary irradiation with 26 Gy in five fractions was non-inferior to standard fractionation, with no increase in brachial plexopathy, lymphoedema, or cardiopulmonary toxicity [19]. Although regional nodal irradiation was not included in the present study, these findings reinforce the broader biological safety of the 26 Gy schedule and underscore the importance of careful patient selection when nodal irradiation or boost strategies are considered.

Notably, in FAST-Forward, tumor-bed boosts were delivered sequentially, and simultaneous integrated boost (SIB) was not evaluated in a randomized setting. Our experience therefore complements the existing evidence by demonstrating the technical feasibility of delivering a modest SIB within an ultra-hypofractionated framework when modern image guidance and intensity-modulated delivery are employed. This observation should be interpreted cautiously and highlights the need for longer follow-up and prospective validation.

### 4.2. Organ Sparing and Late Effects

The safe implementation of ultra-hypofractionated schedules is closely linked to advances in radiotherapy delivery technology. Helical tomotherapy, a rotational image-guided intensity-modulated radiotherapy platform, enables continuous helical delivery, fine beam modulation, and daily image guidance, thereby facilitating excellent dose conformity and homogeneity.

There are concerns regarding low-dose spillage to the heart and lungs when rotational radiotherapy techniques are used. In the present study, strict cardiac constraints (heart V1.5Gy < 30%) were consistently achieved. The mean heart dose of 1.67 Gy observed in left-sided patients is comparable to values reported with deep-inspiration breath-hold techniques in conventionally fractionated regimens, suggesting no increased risk of late cardiac events.

Cardiac dose sparing remains a critical consideration, particularly for left-sided breast cancer. The low mean heart doses observed in this cohort indicate that helical tomotherapy can effectively mitigate cardiac exposure even in ultra-hypofractionated regimens. Furthermore, incorporation of SIB did not compromise dosimetric quality or increase acute toxicity, supporting completion of comprehensive adjuvant radiotherapy within a single week.

The mean contralateral lung V5Gy for the cohort was 3.1%. However, this value was influenced by two outliers (24.4% and 22.2%) associated with medialized breast anatomy. When these two cases were excluded, the V5Gy for the remaining patients ranged from 0.0% to 9.0%, with a median of 0.48%. While HT increases the volume of normal tissue receiving low doses, it provides superior sparing of high-dose regions for the heart and ipsilateral lung. A recent study by Hsu et al. demonstrated that original HT plans for left-sided breast cancer typically show a higher contralateral breast mean dose than tangential partial-arc volumetric modulated arc therapy (t-VMAT) [20]. In clinical practice, the risk of secondary malignancies is frequently mitigated through the use of ‘directional’ or ‘complete’ blocks during optimization to specifically spare the contralateral structures. At our institution, the routine implementation of these blocking techniques is recommended [21,22].

In this study, the use of HT resulted in a mean contralateral breast dose of 3.0 Gy. The characteristic of HT is the wider distribution of low doses to healthy peripheral tissues compared to 3D-conformal radiotherapy. Contralateral breast exposure following RT has been associated with an increased risk of secondary breast cancer. The increased risk of developing cancer has been reported to be dose-dependent, and is particularly evident in younger women who undergo RT before the age of 40–45 years [23,24].

### 4.3. Safety of Simultaneous Integrated Boost (SIB)

The integration of a SIB into a five-fraction regimen remains an evolving area of clinical investigation. While the FAST-Forward trial employed sequential boost delivery, emerging studies—including the HYPORT adjuvant trial [25] and a prospective registered case series reported by Dzhugashvili, M., et al. [15]—have begun to explore SIB in the context of ultra-hypofractionation. In the present study, SIB doses of up to 30 Gy (6 Gy per fraction) were delivered without an increase in acute toxicity.

In contrast to the cohort reported by Mezghani et al., in which Grade 2 acute toxicity occurred in approximately 18.6% of patients [16], only Grade 0–1 toxicity was observed among SIB-treated patients in our study. These findings suggest that with strict image guidance and tomotherapy-based planning, the radiobiological challenges associated with high dose per fraction SIB can be effectively managed without compromising skin integrity.

### 4.4. Treatment Planning Considerations: Tomotherapy and Fraction Size Effects

Advanced treatment planning plays a pivotal role in the safe delivery of ultra-hypofractionated WBI, in which high dose per fraction amplifies the clinical impact of dose heterogeneity and low-dose organ exposure.

Comparative planning studies in accelerated partial breast irradiation have highlighted both strengths and limitations of helical tomotherapy. While target conformity and homogeneity are generally superior to those achieved with three-dimensional conformal radiotherapy, low-dose exposure to the heart and lungs may increase if planning is not carefully optimized [26]. This trade-off underscores the importance of stringent planning constraints, modulation control, and directional blocking, particularly in ultra-hypofractionated regimens.

Our findings are consistent with these observations. With contemporary planning objectives and careful hotspot suppression, helical tomotherapy achieved low mean heart dose and acceptable ipsilateral lung exposure despite the inherently broader low-dose bath. The near absence of high-dose hotspots (V105% and V107%) is clinically relevant, given evidence that ultra-hypofractionated breast irradiation lies on a steep dose–response curve in which even modest increases in dose heterogeneity may result in disproportionately higher toxicity.

A critical consideration in ultra-hypofractionated breast radiotherapy is the management of intrafraction motion and the “interplay effect,” particularly given the high fractional doses of 5.2–6.0 Gy. In this cohort, the average beam-on time was 19.8 min. While this is longer than typical VMAT delivery, evidence suggests that the impact of intrafraction motion during HT remains clinically minor. Ricotti, R., et al. demonstrated that even with extended monitoring times, the maximum respiratory displacement and baseline drift in breast HT are typically <2 mm [27], which is well within our 5–6 mm PTV margins. Furthermore, the “interplay effect”—the interference between target motion and the dynamic movement of the multi-leaf collimator (MLC)—is effectively mitigated in HT through a statistical “blurring” or “smearing” phenomenon. Unlike step-and-shoot IMRT, the helical nature of HT involves multiple gantry rotations and dozens of projections per fraction, which averages out instantaneous dose deviations across the breathing cycle [28]. Consequently, while our homogeneity index values are based on static planning, the radiobiological impact of motion-induced dose uncertainty is likely minimal, supporting the safety of this 5-fraction schedule.

Beyond planning technique, fraction size itself is a critical determinant of toxicity and cosmesis. The systematic review and meta-analysis evidence indicates that ultra-hypofractionation provides oncologic outcomes comparable to longer regimens, with similar rates of grade ≥ 2 acute dermatitis, although long-term toxicity data remain limited [17]. Contemporary clinical series using advanced modulated techniques, such as hybrid volumetric modulated arc therapy, have further demonstrated that ultra-hypofractionated WBI with or without SIB can be delivered with acceptable acute toxicity and preserved esthetic outcomes [29]. Our tomotherapy-based experience complements these reports and reinforces the concept that treatment technique selection and planning quality are as important as fractionation schedule in determining clinical tolerance.

### 4.5. Acute Skin Toxicity and Biological Considerations

Acute skin toxicity remains a key concern influencing clinician adoption of ultra-hypofractionated regimens. In the present study, acute dermatitis was predominantly mild, consistent with both randomized trial data and real-world clinical experience [16,17,30]. Meta-analysis evidence further suggests no statistically significant increase in acute or late toxicity with ultra-hypofractionation compared with moderate hypofractionation. While the FAST-Forward trial reported Grade 2 acute skin toxicity in approximately 10–15% of patients, no Grade ≥ 2 toxicity was observed in our cohort.

This favorable toxicity profile may be partly attributable to the use of helical tomotherapy rather than three-dimensional conformal radiotherapy. Three-dimensional conformal techniques are more likely to generate hotspots exceeding 107% in regions such as the lateral breast or inframammary fold, which correlate with acute desquamation. In contrast, the intensity modulation achievable with tomotherapy effectively suppresses these hotspots (V107% is 0–0.4% in the present study), thereby reducing the risk of skin injury even at high fractional doses.

From a biological perspective, radiation-induced skin dermatitis (RISD) arises from complex inflammatory and oxidative stress pathways involving keratinocyte injury, endothelial dysfunction, and cytokine-mediated tissue remodeling. These mechanisms, including the roles of reactive oxygen species and transforming growth factor-β signaling, have been comprehensively described and highlight that toxicity severity is influenced not only by fraction size but also by irradiated volume and dose heterogeneity [31].

The skin reactions and inflammatory responses typically begin within 1 to 4 weeks after initiating RT. The main acute inflammatory phase usually appears around week 2–3 [32,33]. Compared with moderate hypofractionation or conventional regimen, in which skin irradiation persists through the onset of skin injury, the ultra-hypofractionation course is completed within 1 week, with no further radiation dose to aggravate the skin inflammation. This is a theoretical hypothesis to explain the alleviation of acute skin toxicity observed in our cohort.

### 4.6. Limitations

This study has several limitations, including its retrospective design, single-institution setting, relatively small sample size, and short follow-up duration. A limitation of this study is that other toxicities, including breast edema, pain, and cosmetic outcomes, were not evaluated in this cohort. Although acute toxicity was minimal, longer follow-up is required to assess late effects such as fibrosis, telangiectasia, cosmetic outcomes, and durable local control, which typically emerge two to five years after treatment. Finally, we emphasize that these results represent a preliminary institutional experience. Given the small sample size—particularly within the SIB subgroup—and the relatively short follow-up period, these findings should not be interpreted as a replacement for long-term randomized trial data. Nevertheless, the present data provide clinically relevant real-world evidence supporting the safety and effectiveness of ultra-hypofractionated whole-breast irradiation delivered using helical tomotherapy.

## 5. Conclusions

In conclusion, ultra-hypofractionated whole-breast irradiation (26 Gy in five fractions), with or without a simultaneous integrated boost, delivered via helical tomotherapy, is a dosimetrically robust and technically feasible approach. Our preliminary data show excellent acute toxicity profiles and high treatment efficiency. However, given the short follow-up and small cohort, continued long-term monitoring is essential to confirm durable local control and assess late normal tissue effects.

## Figures and Tables

**Table 1:** Patient and Tumor Characteristics (*N* = 40).

Characteristic	Value
Age (years)	Mean ± SD (Range): 55.7 ± 10.8 (35–80)
Laterality, n (%)	
Left	22 (55%)
Right	18 (45%)
Histology, n (%)	
Invasive Ductal Carcinoma (IDC)	23 (57.5%)
Invasive Lobular Carcinoma (ILC)	2 (5%)
Ductal Carcinoma In Situ (DCIS)	12 (30%)
Mucinous Carcinoma	3 (7.5%)
Pathological T Stage, n (%)	
Tis	12 (30%)
T1	24 (60%)
T2	4 (10%)
Tumor size (cm)	Mean ± SD (Range): 1.3 ± 0.6 (0.2–2.5)
Pathological N Stage, n (%)	N0: 40 (100%)
Tumor Grade, n (%)	
Grade 1	16 (40%)
Grade 2	18 (45%)
Grade 3	6 (15%)
Receptor Status, n (%)	
ER positive	34 (85%)
PR positive	33 (82.5%)
HER2 positive	6 (15%)
Systemic Therapy, n (%)	
Chemotherapy	10 (25%)
Hormone therapy	32 (80%)
Anti-HER2 therapy	6 (15%)
Cigarette smoking, n (%)	1 (2.5%)

Abbreviations: SD, standard deviation; IDC, invasive ductal carcinoma; ILC, invasive lobular carcinoma; DCIS, ductal carcinoma in situ; ER, estrogen receptor; PR, progesterone receptor; HER2, human epidermal growth factor receptor 2.

**Table 2:** Dosimetric Parameters (*N* = 40).

Parameter	Mean ± SD (Range)
Target Volumes	
CTV Volume (mL)	503 ± 221.5 (190–1131.0)
PTV Volume (mL)	862.3 ± 285.1 (405.6–1574.0)
Prescription Dose, n (%)	
26 Gy/5 fractions (Whole Breast)	30 (75%)
29–30 Gy/5 fractions (SIB)	10 (25%)
Target Coverage	
PTV V95% (%)	97.8 ± 1.4 (95.0–100.0)
PTV V105% (%)	2.8 ± 1.7 (0.0–5.0)
PTV V107% (%)	0.1 ± 0.1 (0.0–0.4)
PTV Dmax (Gy)	29.2 ± 1.6 (27.7–32.4)
Organs at Risk (OARs)	
Ipsilateral Lung Mean Dose (Gy)	4.1 ± 0.5 (3.0–5.0)
Ipsilateral Lung V8Gy (%)	12.9 ± 2.8 (2.1–14.9)
Whole Lung Mean Dose (Gy)	2.4 ± 0.6 (0.4–3.34)
Heart Mean Dose (Gy)	1.4 ± 0.5 (0.6–3.0)
Heart V1.5Gy (%)	19.9 ± 9.7 (2.0–29.9)
Heart V7Gy (%)	2.0 ± 2.0 (0.0–4.9)
Contralateral Breast Mean Dose (Gy)	3.0 ± 0.7 (1.7–5.0)
Contralateral Lung V5Gy (%)	3.1 ± 5.4 (0.0–24.4)
Areola Dmax (Gy)	27.7 ± 1.4 (26.5–31.3)

Abbreviations: SD, standard deviation; CTV, clinical target volume; PTV, planning target volume; SIB, simultaneous integrated boost.

**Table 3:** Comparison of Dosimetric Parameters for Whole Breast RT or SIB.

Parameter	Whole Breast RT (26 Gy)	SIB (29–30 Gy)
PTV V95% (%)	97.9	97.4
PTV V105% (%)	2.8	2.9
PTV V107% (%)	0.09	0.1

Abbreviations: PTV, planning target volume; RT, radiotherapy; SIB, simultaneous integrated boost.

**Table 4:** Clinical Outcomes and Toxicity (*N* = 40).

Outcome	Value
Follow-up Duration (months)	
Median (Range)	14.8 (1.1–22.7)
Acute Skin Toxicity (CTCAE), n (%)	
Grade 0	20 (50%)
Grade 1	20 (50%)
Grade ≥ 2	0 (0.0%)
Local Control, n (%)	
Local Recurrence	0 (0.0%)
Local Control Rate	100%
Survival, n (%)	
Overall Survival	40 (100%)
Cancer-Specific Survival	40 (100%)

Abbreviations: CTCAE, Common Terminology Criteria for Adverse Events.

## Data Availability

The data presented in this study are available on request from the corresponding author. The data are not publicly available due to patients’ privacy and medical ethics.

## Supplementary Materials

The following supporting information can be downloaded at https://www.mdpi.com/article/10.3390/cancers18061015/s1. Table S1: Dose Constraints for Target Volume and Organs at Risk.
