# Supplementary material for: Ultra-Hypofractionated Whole-Breast Irradiation With or Without Simultaneous Integrated Boost Using Helical Tomotherapy for Early-Stage Breast Cancer: A Real-World Dosimetric and Clinical Outcome Study
Source: Cancers (Basel). 2026 Mar 20;18(6):1015. doi: 10.3390/cancers18061015 (PMC13025570; doi:10.3390/cancers18061015)
Supplement: Supplementary file 1 [file cancers-18-01015-s001.zip › cancers-4180303-supplementary.pdf]

Supplementary Table S1. Dose Constraints for Target Volume and Organs at Risk.

**Structure**	**Parameter**	**Constraint**
Target Volume		
PTV	V95%	>95%
	V105%	<5%
	V107%	<2%
	Dmax	<110%
Organs at Risk (OARs)		
Ipsilateral lung	V8Gy	<15%
Contralateral lung	V5Gy	<10%
Heart	V7Gy	<5%
	V1.5Gy	<30%
Contralateral breast	Dmean	ALAP
Areola	Dmax	≤108%

Abbreviations: PTV, planning target volume; ALAP, As Low As Possible.
